# Support- and meaning-focused coping as key factors for maintaining adult quality of life during the COVID-19 pandemic in Germany

**DOI:** 10.3389/fpubh.2023.1196404

**Published:** 2023-06-12

**Authors:** Caroline Cohrdes, Rüdiger Pryss, Harald Baumeister, Sophie Eicher, Nina Knoll, Heike Hölling

**Affiliations:** ^1^Mental Health Research Unit, Department of Epidemiology and Health Monitoring, Robert Koch Institute, Berlin, Germany; ^2^Institute of Clinical Epidemiology and Biometry, University of Würzburg, Würzburg, Germany; ^3^Department of Clinical Psychology and Psychotherapy, Ulm University, Ulm, Germany; ^4^Division of Health Psychology, Department of Education and Psychology, Freie Universität Berlin, Berlin, Germany

**Keywords:** WHOQOL-BREF, quality of life, Brief COPE, coping, moderation, sociodemographic characteristics, public health promotion, COVID-19

## Abstract

**Introduction:**

During the COVID-19 pandemic, questions about both consequences and helpful strategies to maintain quality of life (QoL) have become increasingly important. Thus, the aim of this study was to investigate the distribution of coping factors during the COVID-19 pandemic, their associations with QoL and the moderating role of certain sociodemographic characteristics.

**Methods:**

Analyses were based on cross-sectional self-reports from German adult participants (*N* = 2,137, 18–84 years, 52.1% female) of the CORONA HEALTH APP Study from July 2020 to July 2021. Multivariate regression analyses were used to predict (a) coping factors assessed with the Brief COPE and (b) QoL assessed with the WHOQOL-BREF while taking measurement time, central sociodemographic, and health characteristics into account.

**Results:**

During the COVID-19 pandemic, German adults mostly pursued problem- and meaning-focused coping factors and showed a relatively good QoL [Mean values (M) from 57.2 to 73.6, standard deviations (SD) = 16.3−22.6], except for the social domain (M = 57.2, SD = 22.6), and with a decreasing trend over time (β from −0.06 to −0.11, *ps* < 0.01). Whereas, escape-avoidance coping was negatively related to all QoL domains (β = −0.35, *p* < 0.001 for psychological, β = −0.22, *p* < 0.001 for physical, β = −0.13, *p* = 0.045 for social, β = −0.49, *p* < 0.001 for environmental QoL), support- and meaning-focused coping showed positive associations with various QoL domains (β from 0.19 to 0.45, *ps* < 0.01). The results also suggested differences in the pursuit of coping factors as well as in the strength of associations with QoL by sociodemographic characteristics. Escape-avoidance-focused coping was negatively associated with QoL levels in older and less educated adults (simple slopes differed at *ps* < 0.001), in particular.

**Conclusions:**

The results demonstrated what types of coping may be helpful to avoid QoL deterioration (i.e., support- and meaning-focused coping) and provide implications for future universal or targeted health promotion (i.e., older or less educated adults who lack social or instrumental support) and preparedness in the face of unknown challenging societal situations similar to that of the COVID-19 pandemic. Cross-sectional trends of enhanced use of escape-avoidance-focused coping and QoL deterioration point toward a need for increased attention from public health and policy.

## 1. Introduction

Based on the initial conceptual work of Lazarus and Folkman (1), individuals pursue distinct types of coping in response to stressful life events, where coping is defined as the (cognitive or behavioral) effort to adapt to adverse situations that are evaluated as personally harmful and exceeding a person's resources. Following this idea, initial coping research has focused on two different types of coping in response to a stressor: *problem-focused* coping, which is the attempt to actively manage or alter the current problem, and *emotion-focused* coping, which is the striving to regulate negative emotions raised by the current problem (1, 2). Later research has extended and complemented the conceptual framework by introducing the so-called *meaning-focused* coping [i.e., positive orientation and effort to find coherence and meaning in the current problem; (3–5)] and *support-focused* coping (i.e., seeking instrumental or emotional support) as a third and fourth type that individuals use to cope with current problems (4, 6). Such kind of four-factor solutions correspond with the original work of Carver (2) and a number of investigations of the Brief Coping Orientation of Problem Experience (Brief COPE) questionnaire (7, 8), as used in the present study.

However, it should be noted that a significant amount of studies found other ways of summarizing the coping factors of the Brief COPE inventory (9–11). In coping research, there are relatively diverse opportunities of summarizing and grouping coping factors of higher order in general, not least because of differing contextual or methodological approaches and measurement instruments (12, 13). Thus, it is questionable if coping efforts in the context of the COVID-19 pandemic are similar to other stressful life events and can be replicated or integrated into existing coping structures.

### 1.1. Coping during the COVID-19 pandemic

During the COVID-19 pandemic, as in other stressful life situations, adaptive coping ability offers the potential to decrease the risk of long-lasting negative consequences on health and wellbeing (14). Results from recent studies indicate that the general population has already been struggling with their psychological (15), social (16), and environmental wellbeing (17) due to COVID-19-related restrictions and long-term consequences on daily life (18). In Germany, after a temporary decrease during the first lockdown, there was an ongoing increasing trend of certain psychopathological symptoms and poor self-rated mental health over time (19). Information on the pursuit and efficacy of coping factors to reduce and prevent adverse effects on the general population in the long run is thus urgently needed (20–22). This becomes particularly apparent when considering that programs aiming at the universal promotion of coping skills are still seldom and rather group-specific (23) even though there is promising evidence on its potential efficacy in terms of population mental health (24).

Studies summarizing past evidence from stressful life events suggest that certain types can be more helpful than others and that people may differ in their situational application of such (25, 26). Across various stressors, coping factors reflecting active and focused efforts toward problem solution (problem-focused) and a positive orientation (meaning-focused) were predominantly associated with better health and wellbeing, whereas escape-avoidance-focused coping (emotion-focused) showed the opposite pattern (27–29). However, exceptional situations do not appear to allow the application of certain coping factors, such as when confronted with unfamiliar and overwhelming situations or lack of control, hampering the effort of problem-focused coping factors that aim to actively solve a problem (5, 27). Accordingly, other coping factors, such as acceptance or positive reappraisal (meaning-focused) and seeking instrumental or emotional support (support-focused), may become even more important for positive health and wellbeing outcomes (5, 27).

Accordingly, individuals who reappraised the lockdown situation as a chance to rest or pursue hobbies, promote a healthy lifestyle (meaning focus), or seek social or instrumental support reported high life satisfaction and psychological wellbeing (14, 30). Furthermore, early observations revealed a less pronounced use of problem-focused coping and a relatively high use of emotion-focused coping during the COVID-19 pandemic (31). As in other stressful situations, emotion-focused coping, which is often operationalized as avoidance or denial, showed associations with decreased psychological wellbeing across different countries (20, 32, 33). However, the majority of these studies have focused on symptoms of psychopathology or psychological wellbeing and other wellbeing domains have received less attention. In the face of the COVID-19 pandemic, financial constraints, and work- and family-related challenges have been identified as additional major issues over long periods, underscoring the importance of considering the burden in the environmental and social life domains as well (34, 35).

### 1.2. Differences in coping based on sociodemographic factors

Regarding age-related differences in general, one pattern that was relatively robust in previous studies constitutes a decline in problem- and support-focused coping with older age (36, 37). Research on age differences in emotion-focused coping, on the other hand, yielded mixed results (37, 38). The inconclusive findings are likely related to different forms of operationalization of emotion-focused coping (12).

From a theoretical perspective, two main mechanisms are considered to explain age-related differences in coping. The first position assumes that coping varies inherently as a function of age (developmental interpretation), and the second position proposes that coping varies based on the type of situations one must face at different stages of life (contextual interpretation) (38, 39). Both theoretical approaches were complemented by the idea that individuals develop a preference for certain coping factors over time that correspond with personality and have proven to be effective in past situations (40). Against this background, an investigation of coping factors in contexts that are neither age-specific nor comparable to previous experiences appear particularly important to provide more insights into age-related aspects, such as during the current COVID-19 pandemic.

Actual findings of the COVID-19 pandemic suggest a higher risk of burden but also more efficient coping of older adults than younger adults due to less stress reactivity (41). Verhage et al. (42) recently identified acceptance and positive reframing as central coping factors among older adults, emphasizing a meaning focus. However, older adults also reported critically following mitigation measures to avoid an infection, which can be interpreted as higher acceptance of medical appropriateness and a problem-focused coping approach (42).

Besides age, individuals' sex has been discussed as a major source of between-person variation in the pursuit of coping factors across various situations (37, 43). As suggested by the *socialization hypothesis* (44), men are assumed to cope more actively and instrumentally, while women are assumed to cope more passively and emotionally. Men and women are also considered to differ in the respective situations with which they must cope (39) and in the biological basis of hormonal activity that explains sex differences in coping behaviors (summarized as “fight-or-flight” for males and “tend-and-befriend” for females; (6). Consistent with theoretical predictions, women most frequently reported more social support-seeking strategies than men (43, 45), and some evidence also suggests a more frequent use of emotion-focused strategies (45, 46). Evidence on sex differences in problem-focused coping is mixed (43, 45). However, because gender roles are constantly progressing, differences in preferences for coping factors might also change and require ongoing investigation (47).

Furthermore, the educational level is associated with health behaviors (48) and subjective perceptions regarding psychological, physical, social, and environmental wellbeing (49, 50). Individuals with higher levels of education may have more competencies, for instance, in terms of stress regulation (51) or help-seeking behavior (52), and greater access to relevant resources, such as health-related knowledge or well-paid jobs (48, 50). However, the role of educational levels for the use and efficacy of coping factors has not yet attracted sufficient scientific attention, particularly in the general population. Currently, increasing evidence suggests that both the educational level and health knowledge affected people's attitudes and behaviors when handling the COVID-19 pandemic (53, 54). Thus, factors and correlates of coping with the COVID-19 pandemic may also differ as a function of people's educational levels and have implications for public health prevention, but require further investigation.

### 1.3. The present study

Evidence on the factors individuals used to cope during the COVID-19 pandemic and associated restriction measures are still fragmentary but very important (22). Currently (May 10th, 2023), the COVID-19 pandemic in Germany is in the stage of leveling off after the sixths wave and has been demanding on people for a long time. Accordingly, the present study addressed the following exploratory questions (E) and hypotheses (H) to draw conclusions for future response and preparedness in similar demanding societal situations:

1) To what extent did people use different coping factors over a period of time characterized by different stages of the COVID-19 pandemic? Based on previous knowledge, we expected a generally high prevalent use of meaning-focused coping (H1) and aimed to explore the use of other coping factors (E1).
2) Did the pursuit of diverse coping factors differ among people grouped by relevant sociodemographic characteristics (age, sex, and educational level)? By considering previous indications of differences in coping, we expected that older age is associated with less problem-focused and support-focused coping, but higher meaning-focused coping (H2), whereas age differences in the use of escape-avoidance-focused coping require further exploration (E2a). Based on previous conflicting results, we aimed to gather further insights into possible differences in the frequency of problem-focused coping efforts between women and men (E2b). In addition, we explored whether individuals with low education may have used escape-avoidance-focused coping more often than those with high education (E2c).
3) What types of coping factors were positively related to wellbeing during the COVID-19 pandemic and may thus offer entry points for the maintenance of wellbeing in the general population? Since research has focused mainly on psychological wellbeing, we have broadened this perspective by adding physical, social and environmental wellbeing as outcomes. After considering findings from studies assessing other critical life events, we expected positive associations between meaning-focused coping and negative associations between escape-avoidance-focused coping and wellbeing (H3), whereas the roles of problem- and support-focused coping are currently unclear and need to be clarified (E3).
4) Did associations between coping factors and wellbeing differ among people stratified by relevant sociodemographic characteristics (age, sex and educational level) during the COVID-19 pandemic? Due to a lack of knowledge, we explored whether certain sociodemographic characteristics are moderators of these associations (E4).

## 2. Materials and methods

### 2.1. Sample and procedure

The present research is part of the CORONA HEALTH App study, an observational app-based survey on psychological and physical health outcomes during the COVID-19 pandemic that started in July 2020 (55). The present analyses will use cross-sectional self-report data collected from July 2020 to July 2021, including a phase of relaxation after the second wave during the summer in 2020 (July till October 2020), intensification of restriction measures to combat the spreading of the COVID-19 pandemic in Germany with the beginning of the third wave (November 2020 till January 2021: partial lockdown with restrictions on social contacts and traveling; Home office directive; closing of stores in the service sector and gastronomy; cancellation of all entertainment and leisure events; January till April 2021: lockdown; additional obligation to wear an FFP-2 mask in all public places and on public transport, proof of a negative Corona test upon entry into Germany), followed by stepwise relaxation of restriction measures and infection rates from April until July 2021. Participation was voluntary and without reimbursement but restricted to adults 18 years and older. Each participant provided informed consent. The CORONA HEALTH App study was conducted in accordance with the German medical products law and the data protection officer of the University of Würzburg, Germany. The procedures used in this study were in accordance with the 1964 Helsinki declaration and its later amendments and was approved by the ethics committee of the University of Würzburg, Germany (No. 130/20-me).

### 2.2. Measures

#### 2.2.1. Coping

Participants answered the German Brief COPE Inventory (2, 56) consisting of two items for each of the fourteen subscales. The instruction was to “please now assess to what extent the following statements apply to your thinking and acting since the beginning of the COVID-19 pandemic.” Each statement was rated on a 4-point rating scale from “I have not been doing this at all” to “I have been doing this a lot.” Although data-driven approaches reinforce the multidimensional conceptualization of coping, avoiding predetermined higher-order factors and using hierarchical factor analyses in every new investigation is recommended (12). Consequently, categorizations of coping differ somewhat in the coping research field with respect to the particular context and a situational or dispositional focus (56, 57). As recommended by Carver (2), Skinner et al. (12), and as performed by Knoll et al. (56), we therefore summarized the 14 subscales (often referred to as “strategies”) to latent factors by performing both exploratory principal component analyses (PCA) with oblique rotation and subsequent confirmatory factor analyses (CFA) with the R package *lavaan* (58) based on random half split samples. A detailed description can be obtained from the Supplementary material 1. The final model with four latent coping factors (i.e., problem-focused, support-focused, escape-avoidance-focused, and meaning-focused) suggested good fit with $\chi_{\left(45\right)}^{2}$ = 2,105.86. *p* < 0.001, CFI = 0.96, TLI = 0.94. RMSEA = 0.05, SRMR = 0.03 and showed great overlap with the original conceptual work of Carver (2) as well as prior studies with German-speaking samples (56, 59). We built coping factors analogous to the method used by Knoll et al. (56) by calculating the mean of subscales, ranging from min = 1 to max = 4. Internal consistencies were α = 0.76 for problem-focused coping (Active Coping and Planning subscales), 0.83 for support-focused coping (Emotional Support and Instrumental Support subscales), 0.73 for escape-avoidance-focused coping (Denial, Substance Use and Self-Blame subscales) and 0.73 for meaning-focused coping (Positive Reframing, Humor, and Accepting subscales).

#### 2.2.2. Quality of life (QoL)

We used the German version of the WHOQOL-BREF questionnaire as an indicator for quality of life (60, 61), a standardized well-established 26-item instrument comprising the four subscales of psychological (e.g., “To what extent do you feel your life to be meaningful?”) physical (e.g., “How satisfied are you with your capacity for work?”), social (e.g., “How satisfied are you with the support you get from your friends?”) and environmental wellbeing (e.g., “Have you enough money to meet your needs?”) answered on a 5-point rating scale. Consistent with the scoring, values were transformed into scales ranging from 0 to 100 with a mean of 50, with higher values indicating better QoL. Internal consistencies were α = 0.85 for psychological QoL, 0.87 for physical QoL, 0.72 for social QoL, and 0.80 for environmental QoL.

#### 2.2.3. Educational level

Participants were asked to indicate their highest educational degree, and answers were categorized into three levels: low (no school-leaving certificate or graduation after 9 years), moderate (vocational baccalaureate diploma or similar), or high (high school graduation) in accordance with the Comparative Analyses of Social Mobility in Industrial Nations Index [CASMIN; (62)].

#### 2.2.4. Health status

We used three items as indicators of the participant's current health status, the presence of a chronic long-term illness [no = 0, yes = 1, Mini European Health Module; (63)], a (lifetime) medical diagnosis of mental disorder (no = 0, yes = 1), and a current or past COVID-19 infection based on a medically proven positive test (no = 0, yes = 1).

#### 2.2.5. Measurement time

The eligible participation in this study amount to a total of 2,137 over the period of 1 year. To address the time of data collection in our analyses, we have aggregated the cross-sectional points in time on a monthly basis and included them as a metric variable in our analyses. The average number of participants by month was *n* = 365 (SD = 296.85).

### 2.3. Statistical analyses

Analyses were performed with R statistics (64). First, we performed multivariate regression analyses with robust standard errors to investigate differences in the use of coping factors. The four latent factors were regressed on the participant's age, sex, educational level, health status (chronic condition, mental disorder, and COVID-19 infection), and measurement time (aggregated by month). Next, we performed a second multivariate regression analysis with robust standard errors to investigate differences in associations between coping factors and the four QoL domains in various German adult population groups. The health status indicators and the measurement time were entered as control variables, and the four coping factors, sociodemographic characteristics, and their interactions were entered as predictors of the four QoL domains. Significant interactions were probed with simple slope analyses using the R statistics *interactions* package (65). Finally, we performed *post-hoc* power analyses for both multivariate regression models with the R statistics *pwr* package (66).

## 3. Results

The present analyses are based on a convenience sample of *N* = 2,156 participants. Inspection of the plausibility of answers (e.g., correspondence between similar items) careless responding (straight-lining and intraindividual response variability) and extreme outliers (Mahalanobis distance, Cook's distance) led to the exclusion of 19 participants. Thus, the final sample consisted of 2,137 participants (52.1% female, 47.3% male, 0.7% transgender; mean age = 40.98, SD = 13.62). Descriptive statistics as presented in Table 1 include male, female and transgender persons. For robust multivariate regression analyses (Tables 2, 3) including investigations of sex interactions, the 15 transgender participants were excluded due to statistical problems associated with the small group size, resulting in *N* = 2,122 participants (47.5% female, 52.5% male; mean age = 41.03 years, SD = 13.23 years). No values were missing, except for five not assignable educational degrees (acquired abroad), which were excluded case wise.

**Table 1:** Sample characteristics of the 2,137 German adult survey participants (July 2020–July 2021).

		**Coping style** ***M*** **(*****SD*****)**				**Quality of life** ***M*** **(*****SD*****)**			
	***N***	**Prob**	**Sup**	**Esc**	**Mea**	**Psy**	**Phy**	**Soc**	**Env**
**Age group**									
18–29	494	2.54 (0.62)	2.22 (0.75)	1.61 (0.55)	2.56 (0.62)	56.2 (21.4)	67.5 (18.4)	57.2 (22.5)	70.5 (16.5)
30–44	828	2.49 (0.63)	2.06 (0.79)	1.41 (0.47)	2.44 (0.60)	60.8 (20.1)	69.0 (19.2)	57.2 (22.0)	73.4 (16.5)
45–59	590	2.51 (0.65)	1.90 (0.67)	1.31 (0.39)	2.36 (0.63)	65.2 (23.8)	68.0 (21.8)	56.2 (23.8)	75.2 (16.6)
60+	225	2.34 (0.64)	1.58 (0.56)	1.24 (0.35)	2.22 (0.61)	61.0 (21.1)	68.3 (20.2)	59.2 (22.1)	77.1 (15.7)
**Sex**									
Female	1,009	2.59 (0.62)	2.17 (0.72)	1.46 (0.48)	2.44 (0.61)	58.2 (20.7)	65.9 (20.2)	58.0 (22.1)	72.8 (16.6)
Male	1,113	2.39 (0.63)	1.81 (0.67)	1.35 (0.47)	2.40 (0.63)	64.3 (21.1)	71.2 (19.7)	56.4 (23.2)	74.6 (15.9)
Transgender	15	2.43 (0.53)	2.20 (0.75)	1.59 (0.57)	2.40 (0.41)	44.7 (16.6)	52.9 (18.8)	48.9 (18.1)	64.0 (13.1)
**Educational level**									
Low	146	2.34 (0.69)	1.91 (0.78)	1.65 (0.63)	2.14 (0.63)	51.2 (22.2)	56.2 (21.5)	51.3 (23.3)	61.2 (19.1)
Moderate	842	2.42 (0.65)	1.90 (0.72)	1.40 (0.49)	2.37 (0.63)	60.2 (21.5)	66.4 (20.9)	56.1 (23.1)	71.8 (16.7)
High	1,141	2.57 (0.60)	2.09 (0.71)	1.38 (0.43)	2.49 (0.60)	62.8 (20.2)	71.3 (18.7)	58.7 (21.9)	76.6 (14.5)
**Chronic condition** ^a^									
Yes	961	2.48 (0.64)	2.00 (0.73)	1.45 (0.51)	2.37 (0.64)	56.0 (22.4)	59.7 (21.8)	53.0 (23.5)	69.6 (17.6)
No	1,176	2.50 (0.62)	2.00 (0.72)	1.38 (0.44)	2.46 (0.60)	65.0 (19.0)	75.4 (15.6)	60.6 (21.3)	76.9 (14.4)
**Mental disorder** ^a^									
Yes	842	2.50 (0.62)	2.13 (0.74)	1.55 (0.54)	2.35 (0.63)	50.4 (21.2)	57.9 (21.0)	50.7 (23.1)	68.3 (20.2)
No	1,263	2.49 (0.64)	1.91 (0.69)	1.30 (0.39)	2.47 (0.61)	68.4 (17.6)	75.5 (15.8)	61.9 (21.0)	77.7 (13.7)
**COVID-19 infection** ^b^									
Yes	85	2.46 (0.61)	2.37 (0.74)	1.68 (0.63)	2.39 (0.63)	52.0 (22.0)	56.2 (21.4)	54.8 (22.6)	67.4 (17.7)
No	2,052	2.49 (0.63)	1.99 (0.72)	1.40 (0.47)	2.42 (0.62)	61.3 (21.0)	68.8 (20.0)	57.3 (22.6)	73.9 (16.2)
Total	2,137	2.49 (0.63)	2.00 (0.72)	1.41 (0.48)	2.42 (0.62)	61.0 (21.1)	68.3 (20.2)	57.2 (22.6)	73.6 (16.3)

^a^Self-reported lifetime diagnosis.

^b^Medically proven positive COVID-19 test.

Prob, problem-focused; Sup, support-seeking-focused; Esc, escape-avoidance-focused; Mea, meaning-focused; Psy, psychological; Phy, physical; Soc, social; Env, Environmental. Coping scales min = 1 and max = 4; quality of life domains min = 0 and max = 100.

**Table 2:** Differences in coping by age, sex, educational level, health status, and measurement time (*N* = 2,122) determined using multivariate regression analysis.

	**Problem-focused coping**				**Support-focused coping**			
	***B***	***SE***	β	***p***	***B***	***SE***	β	***p***
Intercept	**2.48**	**0.09**		**< 0.001**	**2.39**	**0.07**		**< 0.001**
Female vs. male	**−0.20**	**0.03**	**−0.21**	**< 0.001**	**−0.28**	**0.03**	**−0.22**	**< 0.001**
Age	**−0.15**	**0.05**	**−0.16**	**0.001**	**−0.15**	**0.01**	**−0.33**	**< 0.001**
Low vs. moderate education	0.08	0.06	0.05	0.226	−0.01	0.07	−0.01	0.925
Low vs. high education	**0.20**	**0.06**	**0.17**	**0.001**	**0.17**	**0.07**	**0.12**	**0.011**
No vs. yes chronic condition^a^	−0.01	0.03	−0.01	0.944	0.02	0.03	0.01	0.452
No vs. yes mental disorder^a^	0.01	0.03	0.01	0.892	**0.16**	**0.03**	**0.20**	**< 0.001**
No vs. yes COVID-19^b^ infection	−0.06	0.07	−0.02	0.372	**0.25**	**0.08**	**0.08**	**0.002**
Time	−0.01	0.01	−0.02	0.252	0.01	0.01	0.03	0.07416pt
	**Escape-avoidance-focused coping**				**Meaning-focused coping**			
Intercept	**1.78**	**0.07**		**< 0.001**	**2.55**	**0.06**		**< 0.001**
Female vs. male	−0.02	0.02	−0.01	0.366	−0.03	0.03	−0.02	0.268
Age	**−0.12**	**0.01**	**−0.16**	**< 0.001**	−0.03	0.01	−0.05	0.061
Low vs. moderate education	**−0.20**	**0.05**	**−0.14**	**< 0.001**	**0.22**	**0.06**	**0.24**	**0.017**
Low vs. high education	**−0.21**	**0.05**	**−0.21**	**< 0.001**	**0.30**	**0.05**	**0.25**	**< 0.001**
No vs. yes chronic condition^a^	**0.05**	**0.02**	**0.04**	**0.014**	−0.01	0.03	−0.01	0.659
No vs. yes mental disorder^a^	**0.22**	**0.02**	**0.16**	**< 0.001**	**−0.09**	**0.03**	**−0.07**	**< 0.001**
No vs. yes COVID-19 infection	**0.17**	**0.06**	**0.05**	**0.009**	−0.02	0.07	−0.01	0.752
Time	**0.02**	**0.01**	**0.09**	**< 0.001**	−0.01	0.01	−0.03	0.094

^a^Self-reported lifetime diagnosis.

^b^Medically proven positive COVID-19 test.

B, unstandardized coefficients; SE, robust standard errors; β, standardized coefficient.

R^2^ for problem-focused coping was 0.04, for support-focused coping 0.14, for escape-avoidance-focused coping 0.17, and for meaning-focused coping 0.06. Coding of sex: male = 0, female = 1; educational level: low = 0, moderate = 1, high = 2; self-reported chronic condition: no = 0, yes = 1; self-reported diagnosis of a mental disorder: no = 0, yes = 1; COVID-19 infection; no = 0, yes = 1. Significant results at *p* < 0.05 are highlighted in boldface.

**Table 3:** Differences in associations between coping and psychological as well as physical QoL among participants of various age, sex and educational level (*N* = 2,122) and while controlling for health status and measurement time, as calculated using multivariate regression analyses.

	**Psychological QoL**				**Physical QoL**			
	***B***	***SE***	β	***p***	***B***	***SE***	β	***p***
Intercept	**67.88**	**1.85**		**< 0.001**	**74.40**	**2.06**		**< 0.001**
Female vs. male	**2.18**	**0.39**	**0.05**	**0.003**	**1.77**	**0.74**	**0.06**	**0.017**
Age	**2.46**	**0.39**	**0.12**	**< 0.001**	0.01	0.40	< 0.01	0.992
Low vs. moderate education	1.64	1.70	0.03	0.332	**4.93**	**1.91**	**0.13**	**0.010**
Low vs. high education	1.38	1.69	0.04	0.413	**6.34**	**1.88**	**0.18**	**< 0.001**
No vs. yes chronic condition^a^	**−5.17**	**0.71**	**−0.12**	**< 0.001**	**−11.59**	**0.74**	**−0.29**	**< 0.001**
No vs. yes mental disorder^a^	**−10.82**	**0.79**	**−0.25**	**< 0.001**	**−9.81**	**0.81**	**−0.24**	**< 0.001**
No vs. yes COVID-19 infection^b^	0.53	0.57	0.01	0.353	0.02	0.62	0.02	0.971
Time	**−0.55**	**0.12**	**−0.08**	**< 0.001**	**−0.64**	**0.12**	**−0.10**	**< 0.001**
Problem-focused coping	0.57	1.96	0.03	0.770	1.84	2.15	0.16	0.055
Support-focused coping	**3.32**	**1.57**	**0.19**	**0.004**	−0.29	1.66	−0.02	0.860
Escape-avoidance-focused coping	**−7.47**	**1.14**	**−0.35**	**< 0.001**	**−5.21**	**1.23**	**−0.22**	**< 0.001**
Meaning-focused coping	**5.04**	**1.90**	**0.24**	**0.008**	2.09	1.97	0.01	0.290
Problem-focused coping × age	−0.71	0.43	−0.03	0.100	**−1.17**	**0.47**	**−0.11**	**0.006**
Support-focused coping × age	−0.63	0.40	−0.03	0.109	0.04	0.41	< 0.01	0.920
Escape-avoidance-focused coping × age	**−0.83**	**0.41**	**−0.04**	**0.033**	−0.47	0.43	−0.03	0.303
Meaning-focused coping × age	0.70	0.44	0.03	0.111	**1.26**	**0.47**	**0.12**	**0.004**
Problem-focused coping × male	**−1.84**	**0.95**	**−0.06**	**0.036**	−1.46	0.46	−0.06	0.087
Support-focused coping × male	0.49	0.85	0.02	0.563	1.59	0.84	0.04	0.167
Escape-avoidance-focused coping × male	−1.00	0.81	−0.03	0.218	−1.19	0.79	−0.04	0.131
Meaning-focused coping × male	−0.36	0.90	−0.01	0.685	−0.83	0.91	−0.02	0.361
Problem-focused coping × moderate education	1.58	2.06	0.05	0.446	0.57	2.21	0.03	0.797
Problem-focused coping × high education	2.61	2.04	0.09	0.302	0.26	2.17	0.03	0.904
Support-focused coping × moderate education	**−3.22**	**1.68**	**−0.11**	**0.046**	−0.57	1.72	−0.02	0.743
Support-focused coping × high education	**−3.07**	**1.64**	**−0.19**	**0.038**	−0.50	1.68	−0.01	0.764
Escape-avoidance-focused coping × moderate education	−0.21	1.28	−0.01	0.873	< 0.01	1.36	0.01	0.998
Escape-avoidance-focused coping × high education	−0.84	1.24	−0.03	0.498	< 0.01	1.30	0.02	0.233
Meaning-focused coping × moderate education	−1.23	2.01	−0.04	0.539	−2.05	2.67	−0.02	0.443
Meaning-focused coping × high education	−0.89	1.98	−0.03	0.655	−0.94	2.43	−0.03	0.700
*R* ^2^	0.45				0.40			

^a^Self-reported lifetime diagnosis.

^b^Medically proven positive COVID-19 test.

QoL, quality of life; B, unstandardized coefficients; SE, robust standard errors; β, standardized coefficient.

Coding of sex: male = 0, female = 1; educational level: low = 0, moderate = 1, high = 2; self-reported chronic condition: no = 0, yes = 1; self-reported diagnosis of a mental disorder: no = 0, yes = 1; COVID-19 infection; no = 0, yes = 1. Significant results at *p* < 0.05 are highlighted in boldface.

### 3.1. Use of coping factors

On average, participants reported the use of problem- and meaning-focused coping factors most frequently, while escape-avoidance-focused coping was reported least frequently. The results also suggest several differences in the pursuit of coping factors among different German adult population groups (see Table 2). An older age was associated with a less frequent use of coping factors in general, except for meaning-focused coping. Men reported problem- and support-focused coping less frequently than women, and individuals with a low educational level used factors of problem-, support-, and meaning-focused coping less frequently, whereas they reported a more frequent use of escape-avoidance-focused coping than individuals with a moderate or high educational level. In addition, the participant's health status was related to the use of diverse coping factors: The self-reported diagnosis of a mental disorder was related to less use of meaning-focused coping and a more frequent use of escape-avoidance-focused as well as support-focused coping; individuals with a chronic condition more frequently reported escape-avoidance coping; a current or past COVID-19 infection was associated with more escape-avoidance and support-focused coping. The measurement time was unrelated to coping efforts, except for a positive association with escape-avoidance coping. Supplementary Figure 1 in the SUP shows the use of coping factors averaged across time.

Effect sizes for associations between the considered sociodemographic or health characteristics and coping factors were small to moderate, as was the proportion of explained variance, ranging from 4% (problem-focused coping) to 17% (escape-avoidance-focused coping; see Table 2).

### 3.2. Associations between coping factors and quality of life domains

Overall, participants' QoL was relatively high, as suggested by mean values considerably exceeding the average norm value of 50 scheme (60, 61), except for the comparatively lowest value of the social domain (Table 1). Controlling for measurement time, relevant general health and sociodemographic characteristics, coping factors substantially contributed to the explanation of variance in participants' QoL levels. In particular, escape-avoidance-focused coping was relatively strongly associated to poor QoL in all four domains (see Tables 3, 4). Additionally, support-focused coping was moderately related to higher psychological and social QoL levels and meaning-focused coping to higher psychological as well as environmental QoL levels. Coping factors, measurement time, general health and sociodemographic factors explained 45% of the variance in psychological QoL, 40% of the variance in physical QoL, 20% of the variance in social QoL, and 32% of the variance in environmental QoL (Tables 3, 4).

**Table 4:** Differences in associations between coping and social as well as environmental QoL among participants of various age, sex, and educational level (*N* = 2,122) and while controlling for health status and measurement time, as calculated using multivariate regression analyses.

	**Social QoL**				**Environmental QoL**			
	***B***	***SE***	β	***p***	***B***	***SE***	β	***p***
Intercept	**63.24**	**2.38**		**< 0.001**	**76.21**	**1.59**		**< 0.001**
Female vs. male	**−2.80**	**0.96**	**−0.06**	**0.004**	−0.53	0.65	−0.02	0.410
Age	0.59	0.49	0.03	0.232	**1.89**	**0.32**	**0.12**	**< 0.001**
Low vs. moderate education	2.15	2.12	0.05	0.311	**2.97**	**1.47**	**0.09**	**0.043**
Low vs. high education	1.53	2.09	0.03	0.467	**6.08**	**1.42**	**0.19**	**< 0.001**
No vs. yes chronic condition^a^	**−4.85**	**0.94**	**−0.11**	**< 0.001**	**−4.76**	**0.62**	**−0.15**	**< 0.001**
No vs. yes mental disorder^a^	**−7.82**	**1.01**	**−0.17**	**< 0.001**	**−5.01**	**0.68**	**−0.15**	**< 0.001**
No vs. yes COVID-19 infection^b^	0.74	0.79	0.02	0.348	0.10	0.52	0.01	0.846
Time	**−0.44**	**0.15**	**−0.06**	**0.003**	**−0.55**	**0.10**	**−0.11**	**< 0.001**
Problem-focused coping	−0.28	2.53	−0.01	0.912	**3.32**	**1.49**	**0.20**	**0.026**
Support-focused coping	**10.18**	**2.80**	**0.45**	**< 0.001**	1.31	1.49	0.08	0.329
Escape-avoidance-focused coping	**−3.84**	**2.29**	**−0.13**	**0.045**	**−7.97**	**1.02**	**−0.49**	**< 0.001**
Meaning-focused coping	−0.67	2.46	−0.03	0.784	**4.40**	**1.54**	**0.27**	**0.004**
Problem-focused coping × age	−0.38	0.63	−0.02	0.546	−0.49	0.38	−0.03	0.195
Support-focused coping × age	**−1.10**	**0.53**	**−0.06**	**0.038**	−0.40	0.35	−0.02	0.249
Escape-avoidance-focused coping × age	**−1.98**	**0.51**	**−0.09**	**< 0.001**	**−0.86**	**0.37**	**−0.05**	**0.017**
Meaning-focused coping × age	0.78	0.56	0.04	0.164	0.43	0.38	0.03	0.265
Problem-focused coping × male	−1.26	1.31	−0.04	0.334	−1.18	0.83	−0.05	0.154
Support-focused coping × male	0.21	1.18	0.01	0.856	0.50	0.74	0.02	0.500
Escape-avoidance-focused coping × male	−1.28	1.04	−0.04	0.219	−0.20	0.77	−0.01	0.795
Meaning-focused coping × male	0.23	1.16	0.01	0.844	−0.10	0.81	−0.01	0.902
Problem-focused coping × moderate education	−0.97	2.61	−0.03	0.709	−2.68	1.59	−0.11	0.093
Problem-focused coping × high education	0.01	2.60	< 0.01	0.997	**−2.98**	**1.54**	**−0.13**	**0.045**
Support-focused coping × moderate education	**−6.09**	**2.39**	**−0.17**	**0.003**	−0.63	1.58	−0.03	0.689
Support-focused coping × high education	**−5.69**	**2.32**	**−0.18**	**0.006**	−0.17	1.58	−0.01	0.913
Escape-avoidance-focused coping × moderate education	**−3.34**	**2.28**	**−0.10**	**0.034**	**3.15**	**1.19**	**0.13**	**0.008**
Escape-avoidance-focused coping × high education	−2.81	2.26	−0.08	0.074	**3.53**	**1.26**	**0.14**	**0.001**
Meaning-focused coping × moderate education	3.52	2.49	0.10	0.137	−2.10	1.66	−0.08	0.205
Meaning-focused coping × high education	4.24	2.48	0.13	0.069	−1.64	1.58	−0.07	0.298
*R* ^2^	0.20				0.32			

^a^Self-reported lifetime diagnosis.

^b^Medically proven positive COVID-19 test.

QoL, quality of life; B, unstandardized coefficients; SE, robust standard errors; β, standardized coefficient.

Coding of sex: male = 0, female = 1; educational level: low = 0, moderate = 1, high = 2; self-reported chronic condition: no = 0, yes = 1; self-reported diagnosis of a mental disorder: no = 0, yes = 1; COVID-19 infection; no = 0, yes = 1. Significant results at *p* < 0.05 are highlighted in boldface.

Older participants had better psychological and environmental QoL than younger adults, and male participants showed better psychological and physical QoL but worse social QoL than female participants. The educational level was positively associated with physical and environmental QoL, and individuals with high education scored better than those with low education. Moreover, a chronic somatic condition or mental disorder were negatively associated with each of the four QoL domains, whereas a COVID-19 infection was unrelated to QoL. Later measurement time was related to lower QoL levels (Tables 3, 4).

### 3.3. Moderation effects of age, sex, and educational level on the associations of coping factors with QoL

Participants' age moderated associations between several coping factors and QoL domains (Supplementary Figure 2 of the SUP). In particular, the more older adults used escape-avoidance-focused coping, the lower was their psychological, social and environmental QoL. Further probing of interactions with escape-avoidance-focused coping showed that simple slopes significantly differed from zero for young, middle and older age (psychological QoL: intercept = 67.88; slopes for 1 SD below the mean B = −10.24, SE = 0.49, *p* < 0.001; at mean age B = −11.52, SE = 0.45, *p* < 0.001; 1 SD above the mean B = −12.80, SE = 0.63, *p* < 0.001; social QoL: intercept = 63.24; slopes for 1 SD below the mean B = −5.42, SE = 0.65, *p* < 0.001; at the mean age B = −7.56, SE = 0.56, *p* < 0.001; 1 SD above the mean B = −9.69, SE = 0.88, *p* < 0.001; environmental QoL: intercept = 76.21; slopes for 1 SD below the mean B = −5.51, SE = 0.47, *p* < 0.001; at the mean age B = −6.53, SE = 0.40, *p* < 0.001; 1 SD above the mean B = −7.56, SE = 0.60, *p* < 0.001). Similarly, associations between meaning-focused coping and physical QoL were significantly stronger with older age. Simple slopes differed significantly from zero for younger (1 SD below the mean B = 4.67, SE = 0.64, *p* < 0.001), middle-aged (at the mean B = 4.91, SE = 0.45, *p* < 0.001) and older adults (1 SD above the mean B = 5.15, SE = 0.67, *p* < 0.001; intercept physical QoL: 74.40). Moreover, the results suggested an interaction of age with support-focused coping. Although support-focused coping was related to better social QoL in younger and middle-aged adults (intercept = 63.24; slopes for 1 SD below the mean B = 4.82, SE = 0.68, *p* < 0.001; at the mean age B = 2.97, SE = 0.52, *p* < 0.001) it was unrelated for older adults (1 SD above the mean B = 1.12, SE = 0.75, *p* = 0.14).

The sex of participants moderated associations between problem-focused coping and psychological QoL (Supplementary Figure 3 of the SUP). The association between problem-focused coping and psychological QoL was stronger for female as compared to male participants (intercept = 67.88, female B = 6.73, SE = 0.64, *p* < 0.001, male B = 1.98, SE = 0.68, *p* < 0.001).

Furthermore, the educational level of participants moderated associations between support-focused coping as well as escape-avoidance coping and QoL (Supplementary Figure 4 of the SUP). Simple slope analyses revealed that support-focused coping was positively related to psychological (B = 3.90, SE = 0.89, *p* < 0.001), and social QoL (B = 7.35, SE = 1.90, *p* < 0.001) for individuals with a low educational level. In contrast, for individuals with moderate and high educational levels, support-focused coping was unrelated to the respective QoL domains (psychological QoL: intercept = 67.88; slopes for moderate educational level B = −1.28, SE = 1.01, *p* = 0.20; high educational level B = 0.65, SE = 0.60, *p* = 0.28; social QoL: intercept = 63.24; slope for a moderate educational level B = 1.09, SE = 0.83, *p* = 0.19). One exception was a positive association between support-focused coping and social QoL for individuals with a high educational level (B = 3.22, SE = 0.66, *p* < 0.001). In addition, the probing of interactions revealed a stronger relation of problem-focused coping and environmental QoL with lower as compared to moderate and high educational levels (intercept = 76.21; slopes for low educational level B = 3.53, SE = 1.37, *p* < 0.001, moderate educational level B = 1.22, SE = 0.51, *p* < 0.001; high educational level B = 1.55, SE = 0.40, *p* < 0.001).

### 3.4. Power analyses

*Post-hoc* power analyses suggested a power of 1.0 at an alpha = 0.05 for both multivariate regression analyses, as reported in Table 2 (eight numerators and 2,124 denominators of freedom, with *f*^2^ ranging from 0.04 for problem-focused coping to 0.20 for escape-avoidance-focused coping) and Tables 3, 4 (28 numerators and 2,004 denominators of freedom, with *f*^2^ ranging from 0.25 for social QoL to 0.81 for psychological QoL).

## 4. Discussion

The present research aimed to add knowledge on the use and potential benefits of diverse coping factors in German adults facing the COVID-19 pandemic, as well as possible preventive measures and long-term consequences. We extended previous studies by including the general population and potential moderators such as the age, sex and educational level of participants, and thus our study allowed an in-depth investigation into the pursuit of four different coping factors (problem-, support-, meaning-, and escape-avoidance-focused), their associations with four quality of life domains (psychological, physiological, social, and environmental wellbeing) and interactive effects over a considerable period of time from July 2020 to July 2021.

### 4.1. Use of coping factors

During the COVID-19 pandemic, German adults mainly used coping factors characterized by actively addressing the current problem (problem-focus) and by focusing on positive aspects (meaning-focus). This finding is partially consistent with H1 and previous assumptions of a frequent use of meaning-focused coping in general and in situations with low predictability and controllability in particular (5, 27, 67). As addressed by E1, we found that individuals of the present study used problem- and meaning-focused coping to a similar extent, in accord with other studies (18, 68). Considering that problem-focused coping efforts during the COVID-19 pandemic may manifest in following hygiene and contact restriction measures (18, 42), the present findings can be interpreted as corroborating these earlier findings. Similar to the results of other recent studies (18, 68), the pursuit of escape-avoidance-focused coping was comparatively low. Though the use of such strategies turned out to be particularly detrimental to QoL, especially for certain population groups, as described below.

Individuals from various age groups differed in the pursuit of coping factors. As suggested by H2, an older age was associated with a less frequent use of problem- and support-focused coping. Furthermore, with older age, the use of escape-avoidance-focused coping decreased (E2a). This pattern of results has already been observed in other studies (36, 37, 69) and corresponds with *socioemotional selectivity theory* [SST; (70)]. SST and subsequent work from the emotion regulation research field proposes that whenever people's sense of remaining time is limited, such as in older aged individuals, they increasingly value meaningful social relationships, which are often associated with smaller but closer social networks (71), and prioritize hedonic motivations to maintain or enhance positive affect and wellbeing (72). In contrast, younger adults tend to have open-ended time horizons that are frequently associated with larger social networks and seeking to establish new social ties that serve as important future resources (71), and contra-hedonic motivations to maintain or enhance negative affect that is occasionally beneficial, socially appropriate or instrumental in the long term (72), which in turn may lead to a greater pursuit of support-focused coping or escape-avoidance-focused coping, respectively. The negative associations between age and problem-focused coping corresponds with the idea that with older age, the application of coping factors aiming to actively solve critical events is limited due to incremental loss and reduced controllability [e.g., deterioration of the physical health status, death of close others; (73)]. Consequently, increasing age has been related to changes from an *assimilative* to an *accommodative* mode of coping, i.e., a decrease in coping factors characterized by a modification of a particular situation (e.g., active planning as in problem-oriented coping) and an increase in personal adjustment to situational constraints (e.g., acceptance and positive reframing as in meaning-focused coping) (74). In contrast to these prior findings, we did not observe age-related differences in meaning-focused coping. This finding may be an expression of equalization of coping possibilities in the face of pandemic conditions across diverse age groups, but requires further investigation. Since the findings are consistent with already observed general decreases in the number or intensity of coping factors with older age (75), the fact that only meaning-focused coping did not differ by age may also be a sign of a relatively strong pursuit of this coping style among older adults. However, the reduced pursuit of coping is not to be equated with a loss of skills. In contrast, the majority of research indicates improved coping efficiency with older age (76, 77) and can also be seen as an expression of serenity due to greater life experience and overcoming of challenges (75).

We found that female participants used support- and problem-focused factors more often than men when dealing with the COVID-19 pandemic (E2b). Thus, the results partially refute the theoretical considerations [e.g., socialization hypothesis; (44)] but substantiate other prior empirical findings (43), such as that women cope more actively within the limits of the given pandemic by engaging more frequently in protective behaviors to mitigate the spread of the SARS-CoV-2 virus than men (18, 53, 78). A potential next step for future studies is to include specific protective behaviors in the investigation of sex differences in coping with the COVID-19 pandemic or with other naturalistic critical events. Since the COVID-19 pandemic affects both women and men, explanations based on differences in the experience of events (39) can be excluded. Other explanatory approaches suggesting a rather biological (6) or social (44) basis for sex differences cannot be answered by this study and should be addressed in the future, for example, by including questions on gender role or biophysiological parameters.

Our results moreover support prior observations of educational differences in coping with the COVID-19 pandemic (53, 54). As addressed in E2c, individuals with lower educational levels were more likely to use escape-avoidance-focused coping and less likely to use meaning-, problem-, and support-focused coping. Possible explanations are related to insufficient knowledge, competency and (financial as well as social) resources among less educated individuals (53, 54, 79), and that may become particularly evident during the COVID-19 pandemic. Thus, health-related attitudes and knowledge (i.e., literacy) should be considered in future studies in addition to education.

### 4.2. Associations between coping factors and quality of life domains

The findings showed that the more individuals pursued escape-avoidance-focused coping, the lower was their QoL across all four domains, as expected in H3, and as indicated by previous evidence on maladaptive associations of escape-avoidant-focused coping with several health outcomes in general (20, 27, 33) as well as during the COVID-19 pandemic (21, 32, 80). The finding of positive associations between meaning-focused coping and QoL, substantiates its adaptive potential for wellbeing in general (5) and in the face of the current pandemic situation, in particular (21, 30, 68, 81).

In addition, support- and meaning-focused coping were positively related to psychological and social, respectively environmental QoL (E3). As already observed in adolescents and emerging adults (82), connecting with others appears to be of great importance for people's quality of life. At an early stage of COVID-19 pandemic, older adults also emphasized seeking social support as adaptive coping (81) and social capital has been identified as a central factor for stress experience irrespective of age (14, 34).

Regarding the included covariates of health status, results replicated general findings of lower QoL in individuals with somatic or mental disorders (83). A decreasing trend of all QoL domains with later measurement time found in the present study corresponds with other population-based evidence on trends of German (19) as well as other European adult mental health [e.g., Poland; (84)] and, pending further investigation, might be interpreted as long-lasting effects of the challenges associated with the COVID-19 pandemic.

### 4.3. Moderation effects of age, sex, and educational level on the associations between coping factors and QoL

The present findings also showed that the associations between coping factors and QoL domains were moderated by the participant's age, sex and educational level, as addressed in E4. Although support-focused coping was positively related to social QoL in younger and middle-aged adults, this association was not significant for older adults. This indicates how support seeking can be of particular benefit for young to middle-aged adults' social QoL whereas older adults with lower social QoL may either be less in need of support-focused coping or may require other strategies to enhance their social QoL. Older age is generally indicative of less pronounced seeking of social or emotional support due to motivations to maintain a relatively small selection of close social contacts (71) and to coping efficacy (73, 75). In the figurative sense, those younger and middle-aged adults who were seeking social support may have been unable to rely on close others or less efficient in their coping efforts as compared to older adults. Lack of social support has been identified as one major public health concern affecting health and wellbeing in diverse domains (85) and may have become particularly evident in the current pandemic.

In older-aged participants, associations of escape-avoidance-focused coping with psychological, social, and environmental QoL were more negative than in younger-aged participants. Thus, escape-avoidance-focused coping may have either exerted particularly negative effects on older adults or older-aged participants with low psychological, social, and environmental QoL levels may have tended to pursue maladaptive coping. Similar to support-focused coping, the use of escape-avoidance-focused coping usually tends to decrease as the age of people increases due to efficient emotion regulation skills (77). Thus, older adults who do not fit into the regularly observed pattern of enhanced emotion regulation skills may require specific public health attention.

Another finding was that physical QoL levels were most strongly related to meaning-focused coping with older age. With regard to middle-aged adults, this finding may be a sign of positive adaption to the pandemic in terms of a forced pause in a stage of life usually characterized by career and child care (86). COVID-19-related mitigation measures have been related to reduced stress levels, more family time, opportunities to rediscover hobbies, and promote a healthy lifestyle (30) that might be of great benefit for middle-aged adults. For older adults, who commonly have to deal with physical limitations to an increasing extent, it appears more likely that increased physical QoL is related to meaning-focused coping irrespective of the COVID-19 pandemic. Positive health behaviors and attitudes should be considered in future investigations to draw further conclusions.

The found interaction with the sex of participants suggests that the psychological QoL level of women was better when they used problem-focused coping more frequently. This speaks against the assumption of socialization hypothesis (44) and in favor of the current pandemic encouraging women to pursue problem-focused coping more than in other contexts (18, 53, 78). Moreover, it highlights the positive potential of problem-focused coping for achieving good QoL levels in females. Since the direction of association might also be reversed, female adults with good QoL levels might also have pursued more problem-focused coping.

Positive associations between support-focused coping and social QoL were stronger for individuals with a low educational level than for those with a high or moderate educational level. However, the active request for utilization of emotional and instrumental support in the social and societal environment implies its presence, availability, and knowledge as well as awareness of actual needs. Based on previous evidence on associations between low education and a lack of (emotion) regulation competencies, social or instrumental resources, and health literacy (48, 51, 54, 87), one may conclude that support-focused coping can serve as a buffer for such gaps and thereby may counteract QoL losses. It needs to be further evaluated if relevant information on support offers can be advertised more effectively. Moreover, associations between escape-avoidance-focused coping and psychological, social, and environmental QoL were the most negative for individuals with a low educational level. These findings are in line with other reports on risk behaviors among individuals of lower socio-economic status (48, 51) as well as other results on maladaptive coping factors during the COVID-19 pandemic (79). However, the underlying mechanisms of these associations are not yet fully understood and require more information, such as on personality and resilience, certain knowledge or competencies (e.g., health literacy). Relatively low levels of explained variance, particularly for social QoL and problem-focused coping, may also point to so far unconsidered predictors or moderators.

### 4.4. Limitations

Apart from the aforementioned insights, the present study has limitations that should be acknowledged. First, it employs a cross-sectional design that does not allow to draw conclusions on the direction of the identified associations. Although we tested and reported results on the direction of associations from coping factors to QoL, we cannot rule out bidirectional relationships. Second, generalized and retrospective self-reports yield only a salient snapshot of coping effort and should be supplemented in the future by repeated situational interviews in concrete daily life situations. Another aspect that calls for an intraindividual longitudinal perspective on coping in the long term is that the intensity of used strategies may change in the course of the pandemic due to adaption processes (42). Based on theoretical assumptions (1), individuals may also use coping factors in sequence, for example, by initially regulating emotions and then engaging in solving the problem thereafter. Consequently, coping flexibility (26) may play a crucial role in the face of such a dynamic situation as the COVID-19 pandemic (32).

Third, there is no universal gold standard for summarizing hierarchical coping factors and theoretical as well as methodological approaches to coping are still in constant flux (12). The found four-factor structure largely corresponds with other European research using the Brief COPE inventory from before (7, 56, 88) and partly during the COVID-19 pandemic (59). However, the brief COPE is not all-embracing so that other potentially relevant coping strategies or factors were not considered in this study. Moreover, it is questionable to what extent situational adaption of the instruction (“thinking and acting since the beginning of the COVID-19 pandemic”) can be interpreted as either state or trait coping. Additionally, three subscales had to be excluded due to ambiguity (Venting, Behavioral Disengagement, and Self-Distraction) and one subscale (Positive Reframing) was allowed to load on two factors to achieve the best fit for the present factorial structure, which can be interpreted in line with prior findings and criticism on conceptual overlap or exclusive categorization (12, 59, 80). By synthesizing existing evidence on coping structures, Skinner et al. (12) concluded that it may be beneficial to build rather action-oriented categorizations of coping (e.g., proximity seeking) than functional (e.g., problem-focused coping) or topological (e.g., approach coping).

Last, results were based on a convenience sample and the proportions of people with low educational levels, as well as adults aged 60 years or older, were comparatively low. Although smartphones were already used comprehensively at the time, a small number of people might not have had smartphones available, possibly leading to sampling bias. Thus, the generalizability of this work is limited, as the CORONA HEALTH APP study is not representative for the German population structure, and future studies should endeavor to increase the proportion of people with a lower level of education and older age in particular.

## 5. Conclusions

The present findings are in accord with prior observations on coping efforts and associations with QoL during adverse life events to a relatively large extent. Thus, already identified mechanisms seem to hold true also during the COVID-19 pandemic. For instance, escape-avoidance-focused coping was associated with a reduced QoL in various domains, as observed in other challenging situations of life. Apart from that, this study extended other investigations during the COVID-19 pandemic by considering a comprehensive selection of coping, QoL, sociodemographic and health characteristics, and time of data collection. Thereby, the results yielded additional insights into population groups with enhanced risk of reduced QoL and the potentially beneficial role of certain coping factors for these groups with relevant implications for public health promotion and preparedness.

In sum, support- and meaning-focused coping factors seemed to be important in coping with the actual pandemic and maintaining QoL. Hence, in future pandemics or other naturalistic societal crises, efforts should prioritize on ensuring sufficient offers, information, and low-threshold access to social and instrumental support, such as comprehensive and easily understandable informational campaigns, increasing support hotlines, or initiating voluntary neighborhood organizations. Moreover, public health educational campaigns may help avoid maladaptation (i.e., enhanced substance abuse or denial) and promote adaptive coping factors (e.g., positive reframing or acceptance) by, for example, providing specific recommendations and examples on daily mental hygiene and emotion regulation, in terms of universal health promotion. Pending replication and further investigation, these suggestions may be particularly helpful for individuals with low educational levels, older-aged individuals at risk of lack of adaptive emotion regulation skills, or younger individuals at risk of a lack of emotional or instrumental support. In the present sample, these groups showed the greatest potential of benefit from making use of support-focused coping as well as from reducing escape-avoidance focused coping. Overall, an increasing trend of escape-avoidance-focused coping as well as reduced QoL over time point toward long-term developments in the general population that require particular attention.

## Data availability statement

The raw data supporting the conclusions of this article will be made available by the authors, without undue reservation.

## Ethics statement

The studies involving human participants were reviewed and approved by University of Würzburg, Germany. The patients/participants provided their written informed consent to participate in this study.

## Author contributions

CC, RP, and HB devised the CORONA HEALTH APP study conception and design. RP was mainly responsible for the data acquisition and preparation procedures. CC developed the present research questions and methodology with support from SE, NK, and HH. CC performed the data analyses and wrote a first draft of the manuscript. All authors critically revised the manuscript and approved the final version to be published.
