# Supplementary material for: Support- and meaning-focused coping as key factors for maintaining adult quality of life during the COVID-19 pandemic in Germany
Source: Front Public Health. 2023 Jun 12;11:1196404. doi: 10.3389/fpubh.2023.1196404 (PMC10291068; doi:10.3389/fpubh.2023.1196404)
Supplement: Supplementary file 1 [file Data_Sheet_1.PDF]

Supplementary Material

Support- and Meaning-focused Coping as Key Factors for Maintaining Adult Quality of Life during the COVID-19 Pandemic in Germany

Caroline Cohrdes[1], Rüdiger Pryss[2], Harald Baumeister[3], Sophie Eicher[1], Nina Knoll[4], Heike Hölling[1]

*** Correspondence:** Corresponding Author: CohrdesC@rki.de

1 Description and Results of the Factorial Analyses

First, an inspection of inter-item correlations revealed theoretically sound interrelatedness except for problems regarding the Venting and Behavioral Disengagement subscales. Although significantly interrelated (Venting r=.37 p<.001; Behavioral Disengagement r=.23, p<.001), correlations were comparatively low, and items also correlated substantially with the items of other subscales, indicating ambiguity (Venting with both Instrumental Support items at r=.40 and .44, p<.001; Behavioral Disengagement with Self-Blame and Denial at r=.32, respectively .24, p<.001). In addition, the inclusion of Venting and Behavioral Disengagement in parallel analysis led to poor Kaiser-Meyer-Olkin (KMO) adequacy with values <.70. Thus, we removed both subscales from further analyses. Next, the results from the random split half sample parallel analysis suggested that a four-factor solution is appropriate to describe the present data based on an overall KMO criterion of .82 and >.70 for each of the 12 remaining subscales. The results of a subsequently performed PCA with oblique rotation yielded support for the four-factor solution, explaining 60.5% of the total variance. The first factor subsumed the subscales Active Coping and Planning and accounted for 27.5% of the variance, the second factor (Emotional Support and Instrumental Support subscales) accounted for 16.3%, the third factor (Denial, Substance Use and Self-Blame subscales) accounted for 9.1%, and the fourth factor (Positive Reframing, Humor and Accepting subscales) accounted for 7.6%. All subscales significantly placed a load onto the four factors and reached the cutoff criterion of factor loadings >=.40, except for Self-Distraction and Religion (see factor loadings in Table S2), which were removed from further analyses. The present factor solution widely corresponds with the original work of Carver (1997) (Carver, 1997) and results based on other European countries (France as described in (Baumstarck et al., 2017); Sweden as described in (Nahlen Bose et al., 2015) or the United Kingdom as described in (Hastings et al., 2005)), as well as German-speaking samples (Hanfstingl et al., 2021; Knoll et al., 2005). We thus labeled the four factors congruently as problem-focused coping, support-focused coping, escape-avoidance-focused coping and meaning-focused coping.

Next, a CFA was performed with the remaining 10 subscales and the hypothesized four latent coping factors based on maximum likelihood estimation with the R statistics *lavaan* package (Rosseel 2012) and based on the other random split half sample. Factors were allowed to covary with one another. Based on the results of the PCA, the subscales Active Coping and Planning served as indicators for problem-focused coping; Emotional Support and Instrumental Support were indicators for support-focused coping; Denial, Substance Use and Self-Blame were indicators for escape-avoidance-focused coping; and Positive Reframing, Humor and Acceptance were indicators for meaning-focused coping. The results yielded support for the hypothesized model with marginal fit with $\chi^2(45)$=2,185.10, *p*<.001, *CFI*=.89, TLI=*.83, RMSEA*=.09, *SRMR*=.06. Post hoc model modification indices suggested an enhancement of model fit by allowing the subscale Positive Reframing to load on the problem-focused coping factor. Following a conceptual perspective of cognitive emotion regulation, the loading of Positive Reforming on problem-focused coping is also comprehensible. One could argue that a certain amount of purposeful (active) cognitive processing is necessary to find positive meaning in the face of negative experiences (Garnefski et al., 2001). Adding Positive Reframing to problem-focused coping improved the model to a good fit, $\chi^2(45)$=2,121.97, *p*<.001, *CFI*=.96, *TLI*=.93. *RMSEA*=.05, *SRMR*=.03 (see Table S2).

References

Baumstarck, K., Alessandrini, M., Hamidou, Z., Auquier, P., Leroy, T., & Boyer, L. (2017). Assessment of coping: a new french four-factor structure of the brief COPE inventory. *Health Qual Life Outcomes*, *15*(1), 8. https://doi.org/10.1186/s12955-016-0581-9

Carver, C. S. (1997). You want to measure coping but your protocol' too long: Consider the brief cope. *Int J Behav Med*, *4*(1), 92. https://doi.org/10.1207/s15327558ijbm0401_6

Garnefski, N., Kraaij, V., & Spinhoven, P. (2001). Negative life events, cognitive emotion regulation and emotional problems. *Personality and Individual Differences*, *30*(8), 13111327. https://doi.org/http://dx.doi.org/10.1016/S0191-8869%2800%2900113-6

Hanfstingl, B., Gnambs, T., Fazekas, C., Gölly, K. I., Matzer, F., & Tikvić, M. (2021). The Dimensionality of the Brief COPE Before and During the COVID-19 Pandemic. *Assessment*, https://doi.org/10.1177/10731911211052483

Hastings, R. P., Kovshoff, H., Brown, T., Ward, N. J., Espinosa, F. D., & Remington, B. (2005). Coping strategies in mothers and fathers of preschool and school-age children with autism. *Autism*, *9*(4), 377-391. https://doi.org/10.1177/1362361305056078

Knoll, N., Rieckmann, N., & Schwarzer, R. (2005). Coping as a mediator between personality and stress outcomes: A longitudinal study with cataract surgery patients. *European Journal of Personality*, *19*, 229-247. https://doi.org/10.1002/per.546

Nahlen Bose, C., Bjorling, G., Elfstrom, M. L., Persson, H., & Saboonchi, F. (2015). Assessment of coping strategies and their associations with health related quality of life in patients with chronic heart failure: the brief COPE restructured. *Cardiol Res*, *6*(2), 239-248. https://doi.org/10.14740/cr385w

Rosseel , Y. (2012). lavaan: An R Package for Structural Equation Modeling. *Journal of Statistical Software*, *48*(2), 1–36. https://www.jstatsoft.org/v48/i02/

2 Supplementary Tables

Table S1. Inter-Scale Correlations of the Initial 14 Coping Subscales (*N*=2,137)

	1	2	3	4	5	6	7	8	9	10	11	12	13	14
1. Self-Distraction	1	.20**	.37**	.12**	.28**	.17**	.39**	.19**	.35**	.29**	.35**	.07**	.22**	.24**
2. Denial	.20**	1	.12**	.32**	-.09**	-.04	.02	.23**	.16**	.22**	.07**	-.21**	.34**	.13**
3. Emotional Support	.37**	.12**	1	-.01	.28**	.19**	.31**	.08**	.60**	.42**	.35**	.09**	.17**	.24**
4. Behavioral Disengagement	.12**	.32**	.01	1	-.10**	.03	-.14**	.19**	.02	.08**	-.10**	-.05*	.31**	.06**
5. Positive Reframing	.28**	-.09**	.28**	-.10**	1	.35**	.42**	-.06**	.23**	.14**	.39**	.37**	-.08**	.22**
6. Humor	.17**	-.04	.19**	.03	.35**	1	.12**	.05*	.10**	.06*	.13**	.32**	-.04	.05*
7. Active Coping	.39**	.02	.31**	-.14**	.42**	.12**	1	.03	.34**	.22**	.50**	.21**	.04	.23**
8. Substance Use	.19**	.23**	.08**	.19**	-.06**	.05*	.03	1	.11**	.13**	.07**	-.08**	.26**	.04
9. Instrumental Support	.35**	.16**	.60**	.02	.23**	.10**	.34**	.11**	1	.51**	.40**	.04	.26**	.28**
10. Venting	.29**	.22**	.42**	.08**	.14**	.06*	.22**	.13**	.51**	1	.27**	.02	.24**	.21**
11. Planning	.35**	.07**	.35**	-.10**	.39**	.13**	.50**	.07**	.40**	.27**	1	.18**	.15**	.20**
12. Accepting	.07**	-.21**	.09**	-.05*	.37**	.32**	.21**	-.08**	.04	.02	.18**	1	-.12**	.06**
13. Self-Blame	.22**	.34**	.17**	.31**	-.08**	-.04	.04	.26**	.26**	.25**	.15**	-.12**	1	.14**
14. Religion	.24**	.13**	.24**	.06**	.22**	.05*	.23**	.04	.28**	.21**	.20**	.06**	.14**	1

p<.01**. *p*<.05*

Table S2. Results from Principal Component Analysis Including the Remaining 12 Subscales Hypothesized to Load on Four Latent Coping Factors (*N*=1,068, random half split sample)

	Factor loadings on problem-focused coping	Factor loadings on support-focused coping	Factor loadings on escape-avoidance-focused coping	Factor loadings on meaning-focused coping
Self-Distraction	.36	.12	.36	.11
Denial	-.03	-01	**.55**	.02
Emotional Support	-.06	**.84**	-.04	.05
Positive Reframing	.36	.07	-.15	**.47**
Humor	-.06	.03	.08	**.69**
Active coping	**.73**	.01	-.03	-.04
Substance Use	-.02	-.06	**.46**	.05
Instrumental Support	.13	**.65**	.09	-.11
Planning	**.61**	.07	.10	-.04
Accepting	.24	-.03	-.22	**.40**
Self-Blame	.02	.04	**.61**	.00
Religion	.17	.22	.09	.05
Eigenvalues	3.18	1.94	1.09	0.92
Proportion variance	27.53	16.25	09.05	07.64
Cumulative variance	27.53	43.78	52.83	60.47

Notes. Highest factor loading >=.40 are highlighted in boldface for each subscale.

Table S3. Results from Confirmatory Factor Analysis Including 10 Subscales Hypothesized to Load on Four Latent Coping Factors (*N*=1,069, random half split sample)

	Unstand. estimate	*SE*	Stand. estimate	*p*
**Latent factors**				
Problem-focused coping~				
Positive Reframing	0.347	0.031	.411	<.001
Active Coping	0.530	0.025	.689	<.001
Planning	0.556	0.026	.706	<.001
Support-focused coping~				
Emotional Support	0.619	0.027	.741	<.001
Instrumental Support	0.657	0.026	.823	<.001
Escape-avoidance-focused coping~				
Denial	0.342	0.024	.585	<.001
Self-Blame	0.469	0.031	.618	<.001
Substance Use	0.240	0.023	.398	<.001
Meaning-focused coping~				
Positive Reframing	0.378	0.035	.448	<.001
Humor	0.366	0.030	.463	<.001
Accepting	0.540	0.034	.651	<.001
**Covariances**				
Problem-focused coping~~				<.001
Support-focused coping	0.650	0.032	.650	<.001
Escape-avoidance-focused coping	0.155	0.049	.155	<.001
Meaning-focused coping	0.373	0.051	.373	<.001
Support-focused coping~~				<.001
Escape-avoidance-focused coping	0.393	0.043	.393	<.001
Meaning-focused coping	0.139	0.048	.139	<.001
Escape-avoidance-focused coping~~				<.001
Meaning-focused coping	-0.375	0.051	-.375	<.001
**Variances**				
Positive Reframing	0.351	0.024	.493	<.001
Active Coping	0.311	0.020	.526	<.001
Planning	0.310	0.021	.501	<.001

Emotional Support	0.314	0.024	.451	<.001
Instrumental Support	0.206	0.024	.323	<.001
Denial	0.224	0.015	.657	<.001
Self-Blame	0.357	0.027	.619	<.001
Substance Use	0.307	0.015	.842	<.001
Humor	0.491	0.026	.786	<.001
Accepting	0.396	0.033	.576	<.001

Note. Model fit indices: $\chi^2(45)$=2,121.97. p<.001, *CFI*=.96, *TLI*=.93. *RMSEA*=.05, *SRMR*=.03.

2.1 Supplementary Figures

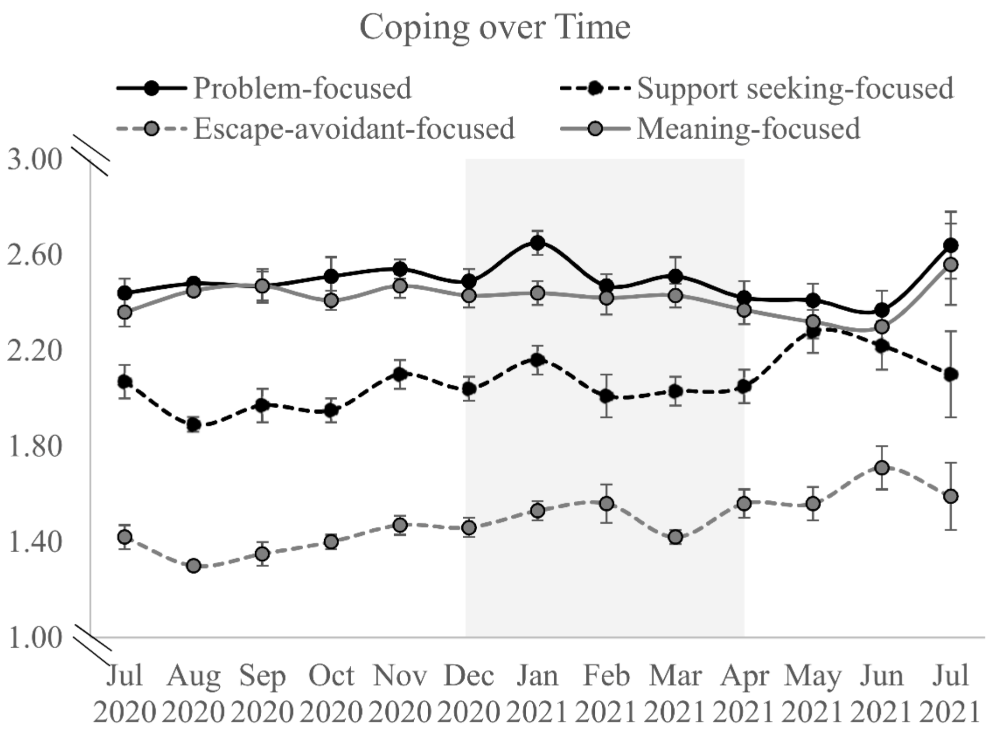

Figure S1. Monthly averaged use of the four coping styles from July 2020 to July 2021. Lockdown period (end of wave 2, before beginning of wave 3) is highlighted in light grey color. Results from robust multivariate regression analysis indicates a significant increase in escape-avoidance focused coping over time (Table 2 in the main text). The average number of participants by month was *n*=365 (*SD*=296.85).

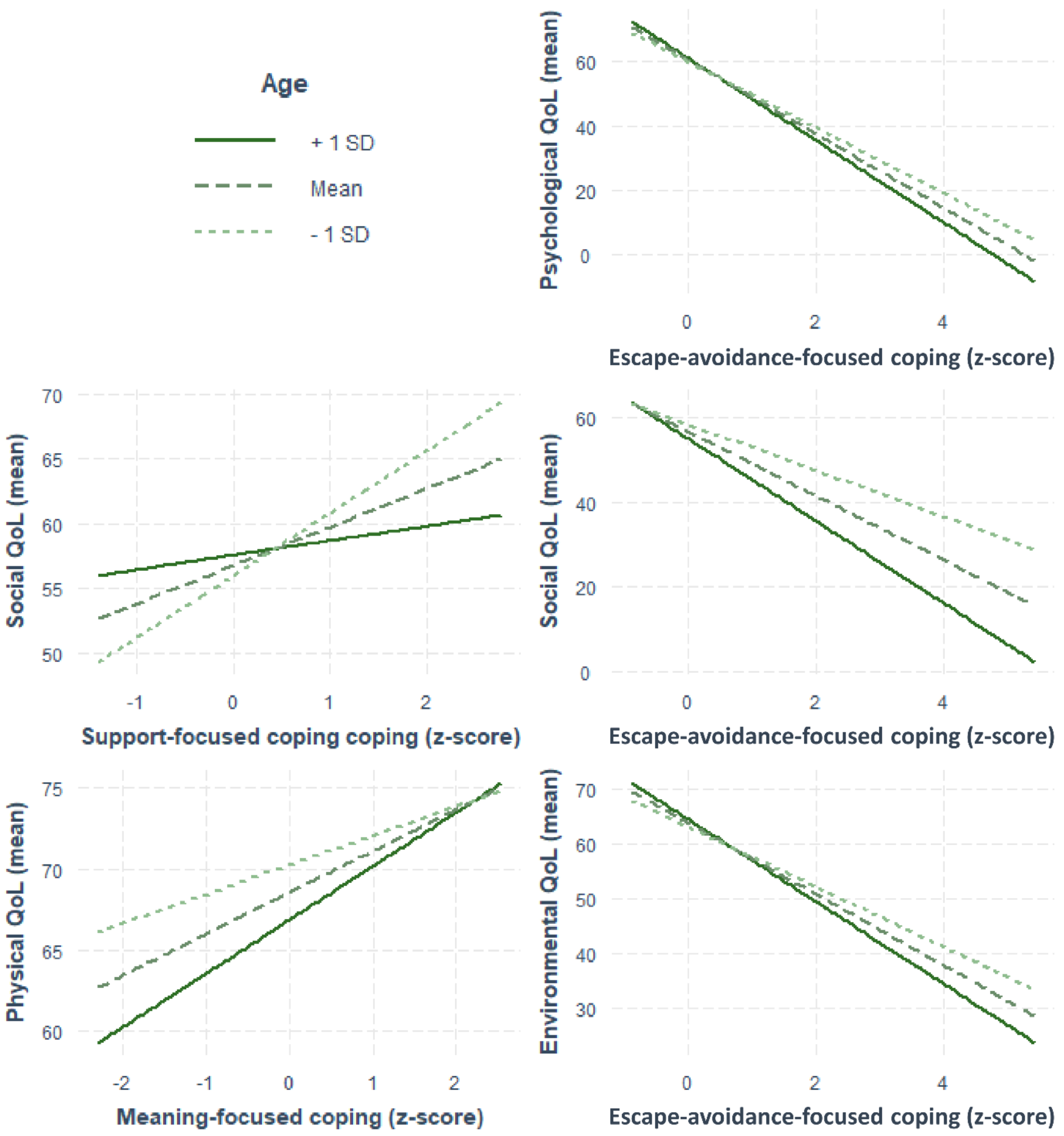

Figure S2. Moderating effects of age (-1*SD*=27.4, *M*=41.03, +1*SD*=54.66) on the associations between coping factors and quality of life domains.

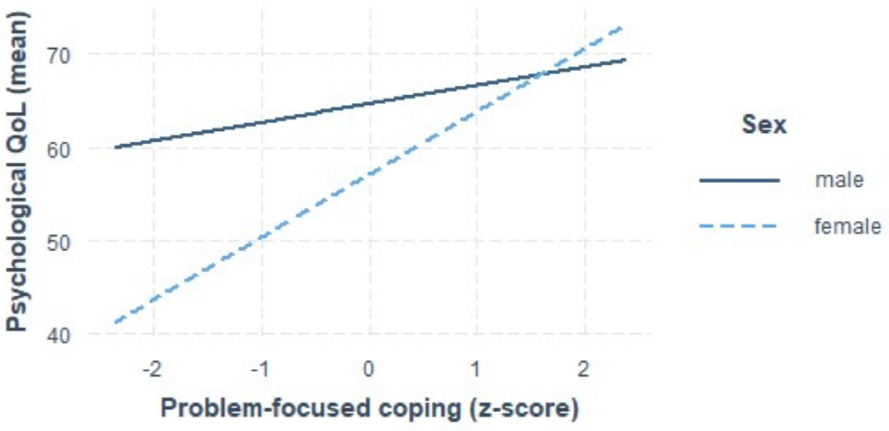

Figure S3. Moderating effects of sex (female and male) on the association between problem-focused coping and psychological quality of life .

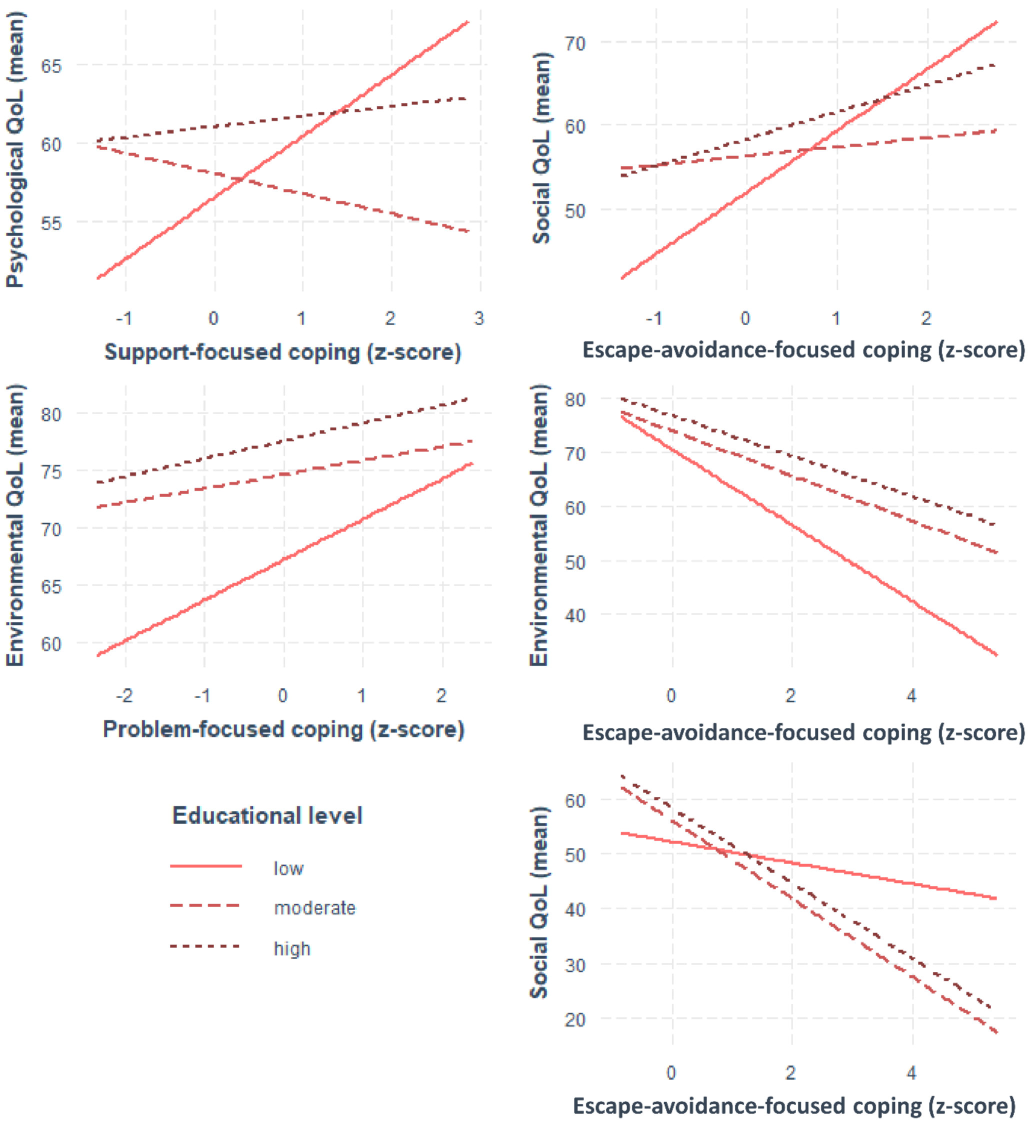

Figure S4. Moderating effects of educational level (low, moderate, and high) on the associations between coping factors and quality of life domains.
